# Supplementary material for: Molecular Structure of Gaseous Oxopivalate Co(II): Electronic States of Various Multiplicities
Source: Int J Mol Sci. 2023 Aug 25;24(17):13224. doi: 10.3390/ijms241713224 (PMC10487729; doi:10.3390/ijms241713224)
Supplement: Supplementary file 1 [file ijms-24-13224-s001.zip › ijms-2539419-supplementary.pdf]

SUPPLEMENTARY MATERIALS

Molecular Structure of Gaseous Oxopivalate Co(II): Electronic States of Various Multiplicities

Nina I. Giricheva[1], Valery V. Sliznev[2], Andrey S. Alikhanyan[3], Ekaterina A. Morozova[3], Georgiy V. Girichev[2,*]

[1] Nanomaterial Research Institute, Ivanovo State University, Ermak str. 39, 153025 Ivanovo, Russia
[2] Department of Physics, Ivanovo State University of Chemistry and Technology, Sheremetevsky ave. 7, 153000 Ivanovo, Russia
[3] Kurnakov Institute of General and Inorganic Chemistry RAS, Leninsky prospect 7, 119991 Moscow, Russia

* Correspondence: g.v.girichev@mail.ru; Tel.: +7-4932-35-98-74

CONTENT

1. Table S1. Coordinates of $Co_4O(piv)_6$ optimized structure by DFT/B3LYP/cc-pVTZ method, M=13……………………………………………………………………………………………………2
2. Table S2. Vibrational frequencies of $Co_4O(piv)_6$ optimized structure by DFT/B3LYP/cc-pVTZ method, M=13. $C_{30}H_{54}Co_4O_{13}$, Number of atoms N=101. ………………………………………………………………. 4
3. Figure S1. Structure of $Co_4O(piv)_6$ complex with numbering atoms ………………………………………..6
4. Table S3. Coordinates of refinement GED structure, Rf=4.5% …………………..…………………………..7
5. Table S4. The results of LS-analisys of GED data. Calculated and experimental amplitudes of vibrations and their groups. ……………………………………………………………………………………….…9

Table S1. Coordinates of $Co_4O(piv)_6$ optimized structure by DFT/B3LYP/cc-pVTZ method, M=13

8	0.000000000	0.000000000	-0.010744000
27	0.000000000	1.870959000	-0.671921000
8	-1.600360000	2.215390000	-1.771654000
8	0.008224000	3.144072000	0.838061000
8	1.602523000	2.220035000	-1.773207000
27	-1.620298000	-0.935480000	-0.671921000
8	-2.723868000	0.277808000	-1.773207000
8	-1.118404000	-2.493647000	-1.771654000
8	-2.726958000	-1.564914000	0.838061000
27	0.000000000	0.000000000	1.976497000
8	-1.604788000	-0.918216000	2.665358000
8	1.597592000	-0.930680000	2.665358000
8	0.007196000	1.848895000	2.665358000
27	1.620298000	-0.935480000	-0.671921000
8	1.121345000	-2.497843000	-1.773207000
8	2.718763000	0.278257000	-1.771654000
8	2.718734000	-1.579158000	0.838061000
6	-2.581592000	-1.479061000	2.089376000
6	2.571701000	-1.496194000	2.089376000
6	2.575032000	1.488848000	-2.110243000
6	0.001864000	-2.974467000	-2.110243000
6	0.009891000	2.975255000	2.089376000
6	-2.576896000	1.485619000	-2.110243000
6	3.676360000	2.093117000	-2.995832000
6	5.011084000	1.989272000	-2.232698000
6	3.376926000	3.557400000	-3.331589000
1	2.434978000	3.658757000	-3.868937000
1	3.310770000	4.167494000	-2.431772000
1	4.174191000	3.958665000	-3.959544000
1	4.982018000	2.565808000	-1.306464000
1	5.818168000	2.388096000	-2.849199000
1	5.241920000	0.954679000	-1.985916000
6	-0.025487000	-4.230379000	-2.995832000
6	1.392336000	-4.703203000	-3.331589000
6	-0.786433000	-3.884149000	-4.290276000
1	-1.797935000	-3.546886000	-4.071954000
1	-0.275354000	-3.099310000	-4.850591000
1	-0.844445000	-4.766869000	-4.928995000
1	1.951088000	-3.938132000	-3.868937000
1	1.341209000	-5.594288000	-3.959544000
1	1.953770000	-4.950958000	-2.431772000
6	3.642238000	-2.122055000	2.997580000
6	4.219445000	-1.008005000	3.892034000
6	4.761742000	-2.765382000	2.173318000
1	4.378910000	-3.555238000	1.528486000
1	5.260439000	-2.034030000	1.538713000
1	5.505055000	-3.200536000	2.843476000
1	4.707532000	-0.235659000	3.295098000
1	4.965494000	-1.429471000	4.567545000
1	3.438205000	-0.538963000	4.487163000
6	-3.658873000	-2.093243000	2.997580000
6	-4.775762000	-2.741099000	2.173318000
6	-4.237549000	-0.967975000	3.877096000

1	-3.458180000	-0.495472000	4.471983000
1	-4.719511000	-0.200222000	3.269339000
1	-4.988966000	-1.379907000	4.552537000
1	-5.268381000	-2.014628000	1.528486000
1	-5.524274000	-3.167250000	2.843476000
1	-4.391742000	-3.538659000	1.538713000
6	-3.650872000	2.137263000	-2.995832000
6	-4.228302000	3.345090000	-2.232698000
6	-4.769261000	1.145804000	-3.331589000
1	-4.386066000	0.279374000	-3.868937000
1	-5.264541000	0.783464000	-2.431772000
1	-5.515400000	1.635623000	-3.959544000
1	-4.713064000	3.031650000	-1.306464000
1	-4.977236000	3.844633000	-2.849199000
1	-3.447736000	4.062296000	-1.985916000
6	0.016635000	4.215298000	2.997580000
6	0.014020000	5.506481000	2.173318000
6	1.280483000	4.153813000	3.877096000
1	1.299999000	3.242608000	4.471983000
1	2.186358000	4.187328000	3.269339000
1	1.299449000	5.010524000	4.552537000
1	0.889471000	5.569866000	1.528486000
1	0.019218000	6.367786000	2.843476000
1	-0.868698000	5.572689000	1.538713000
6	3.756989000	1.261004000	-4.290276000
1	2.821759000	1.311191000	-4.850591000
1	4.550452000	1.652124000	-4.928995000
1	3.970661000	0.216386000	-4.071954000
6	-0.782782000	-5.334362000	-2.232698000
1	-1.794184000	-5.016975000	-1.985916000
1	-0.840932000	-6.232730000	-2.849199000
1	-0.268954000	-5.597458000	-1.306464000
6	-2.982680000	-3.150144000	3.892034000
1	-2.557853000	-3.959013000	3.295098000
1	-2.185859000	-2.708092000	4.487163000
1	-3.720705000	-3.585509000	4.567545000
6	-1.236764000	4.158149000	3.892034000
1	-1.252347000	3.247055000	4.487163000
1	-1.244789000	5.014979000	4.567545000
1	-2.149679000	4.194672000	3.295098000
6	2.957066000	-3.185838000	3.877096000
1	2.533153000	-3.987106000	3.269339000
1	3.689517000	-3.630618000	4.552537000
1	2.158181000	-2.747136000	4.471983000
6	-2.970555000	2.623146000	-4.290276000
1	-2.546404000	1.788119000	-4.850591000
1	-3.706008000	3.114745000	-4.928995000
1	-2.172726000	3.330500000	-4.071954000

Table S2. Vibrational frequencies of $Co_4O(piv)_6$ optimized structure by DFT/B3LYP/cc-pVTZ method, M=13. $C_{30}H_{54}Co_4O_{13}$, Number of atoms N=101.
Number of vibrational degrees of freedom 3N-6 = 297

	ν	ν	ν	ν	ν	ν
1	9.4401	241.2725	601.8118	1050.2429	1460.962	3029.0234
2	10.5008	252.0029	601.9595	1050.2853	1480.2984	3029.0549
3	10.5009	252.0031	607.9097	1050.2853	1480.3334	3029.0746
4	11.2124	255.9229	608.2249	1053.4989	1480.3334	3029.0746
5	11.5346	256.3899	608.2252	1053.4989	1480.4759	3029.2031
6	11.5348	256.3899	609.901	1053.6081	1480.4759	3037.2056
7	14.8747	260.6959	609.9011	1053.7503	1480.4956	3037.2056
8	14.8759	260.8414	612.0895	1053.7503	1487.9117	3037.2562
9	16.415	260.8414	612.0901	1053.8824	1487.9117	3037.2776
10	21.9352	262.6472	612.3607	1226.2437	1488.23	3037.2776
11	22.0387	265.1312	617.9989	1226.5104	1488.6493	3037.5837
12	22.0389	265.1312	788.3352	1226.5272	1488.6493	3084.7869
13	51.4237	265.8227	788.3396	1226.5272	1489.007	3084.7869
14	57.9032	266.6167	788.3396	1226.6818	1492.8761	3084.7897
15	57.9035	266.6168	789.7974	1226.6818	1493.8712	3084.9808
16	61.1074	276.8479	789.7974	1244.7495	1493.8712	3085.0737
17	65.9787	286.2416	789.8023	1244.7495	1494.0806	3085.0737
18	65.9789	286.6701	794.5642	1245.1564	1494.4544	3086.331
19	68.3619	286.6701	794.5643	1245.3652	1494.4544	3086.331
20	68.4187	289.04	795.7289	1245.3652	1501.758	3086.4075
21	68.4197	289.04	795.7369	1246.8433	1502.0373	3086.4075
22	100.7291	289.1073	795.737	1255.2799	1502.0373	3086.4295
23	100.7292	308.2435	798.4783	1255.2799	1502.1195	3086.645
24	102.826	308.2534	897.707	1255.9465	1502.2707	3097.3664
25	105.0801	308.2535	897.707	1255.9465	1502.2707	3097.3664
26	105.0805	310.1104	898.8528	1256.2248	1504.0054	3097.4031
27	105.4514	310.6823	898.8689	1259.5298	1504.0054	3097.5464
28	110.3084	310.6823	898.869	1396.7412	1504.1816	3097.5464
29	110.3085	379.8162	902.3147	1397.0145	1504.2483	3097.7198
30	115.9016	379.8773	939.5371	1397.0145	1504.2483	3109.9986
31	128.0596	379.8773	939.6082	1397.0588	1504.5286	3109.9986
32	128.0602	380.404	939.6082	1397.1842	1526.1417	3110.0384
33	130.7057	380.4702	939.6243	1397.1842	1526.1417	3110.1684
34	132.7889	380.4702	939.6816	1397.7045	1526.6296	3110.185
35	132.7894	402.4576	939.6816	1397.7045	1526.6721	3110.185
36	167.8488	402.4577	946.4181	1398.1085	1526.6721	3113.0326
37	175.5341	402.5455	946.4181	1398.1085	1528.1872	3113.1128
38	177.2206	402.8365	946.5291	1398.2656	1543.0511	3113.1247
39	177.2213	403.0881	946.5546	1399.9881	1543.0511	3113.1247
40	182.5779	403.0882	946.5546	1413.7784	1543.0824	3113.1716
41	182.5792	443.0984	946.6229	1413.7784	1607.9443	3113.1716
42	183.7055	443.0984	971.2591	1414.397	1608.5336	3116.6765
43	210.5447	443.2408	971.4625	1414.4199	1608.5337	3116.6765
44	211.0873	443.4976	971.4625	1414.4199	3027.3637	3116.7272
45	211.0873	446.084	971.6028	1417.4045	3027.3707	3116.8336
46	211.7311	446.084	971.6028	1453.0395	3027.3707	3116.8336

47	211.7311	486.3217	971.6813	1453.0395	3027.5038	3116.9869
48	211.7642	488.6457	1050.0327	1453.9298	3027.6074	
49	240.409	488.6491	1050.1905	1453.9298	3027.6074	
50	240.4095	601.8117	1050.1905	1454.496	3029.0234	

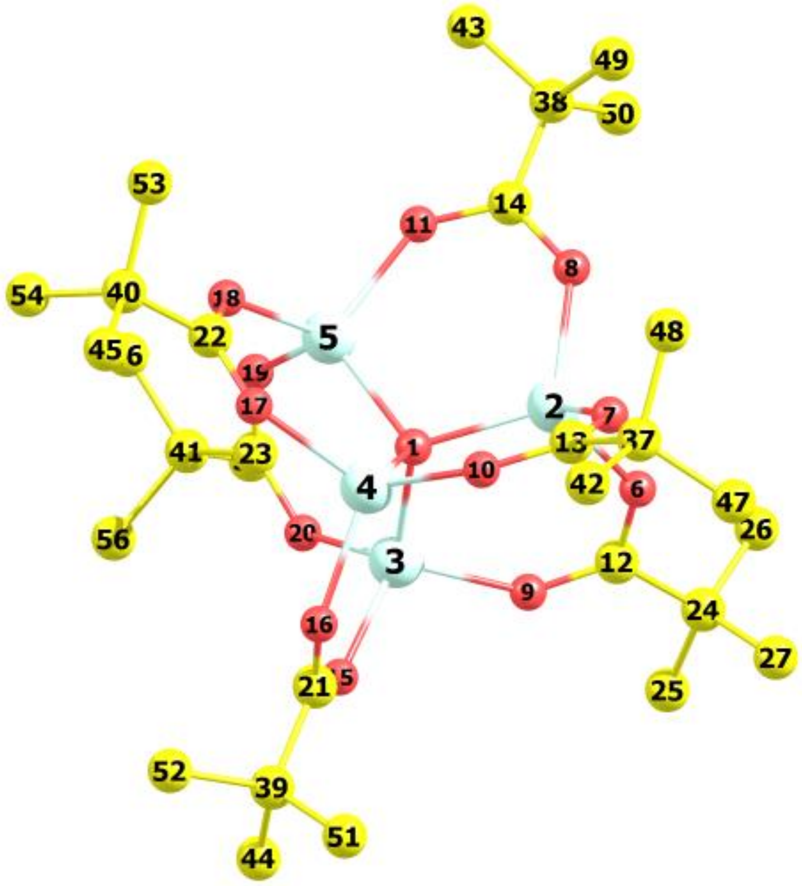

Fig.S1. Structure of $Co_4O(piv)_6$ complex with numbering atoms

Table S3. Coordinates of refinement GED structure, Rf=4.5%

8	0.000000000	0.000000000	0.000000000
27	0.000000000	0.000000000	1.975100000
27	1.848100000	0.000000000	-0.688200000
27	-0.924000000	-1.600500000	-0.688200000
27	-0.924000000	1.600500000	-0.688200000
8	1.822800000	-0.006700000	2.703800000
8	-0.917200000	-1.575200000	2.703800000
8	-0.905600000	1.581900000	2.703800000
8	3.034600000	-0.008200000	0.878200000
8	-1.524400000	-2.624000000	0.878200000
8	-1.510200000	2.632200000	0.878200000
6	2.958000000	-0.010000000	2.140600000
6	-1.487600000	-2.556700000	2.140600000
6	-1.470400000	2.566700000	2.140600000
8	2.209500000	-1.560700000	-1.826200000
8	0.221600000	-2.711400000	-1.829700000
8	-2.456300000	-1.133100000	-1.826200000
8	-2.458900000	1.163800000	-1.829700000
8	0.246800000	2.693800000	-1.826200000
8	2.237400000	1.547600000	-1.829700000
6	1.431300000	-2.505000000	-2.145800000
6	-2.885000000	0.012900000	-2.145800000
6	1.453700000	2.492100000	-2.145800000
6	4.194500000	-0.015800000	3.053800000
6	5.499500000	0.112700000	2.261400000
6	4.102100000	1.173900000	4.029200000
6	4.232700000	-1.343100000	3.836300000
1	6.355900000	0.106200000	2.920400000
1	5.538800000	1.010300000	1.665600000
1	5.625900000	-0.674700000	1.535900000
1	4.947500000	1.158500000	4.702100000
1	3.205500000	1.153700000	4.627600000
1	4.115700000	2.129600000	3.525100000
1	5.077300000	-1.343300000	4.510400000
1	3.338800000	-1.511700000	4.414900000
1	4.314600000	-2.206200000	3.191200000
6	-2.111000000	-3.624700000	3.053800000
6	-2.083600000	3.640500000	3.053800000
6	1.997800000	-3.601600000	-3.062000000
6	-4.117900000	-0.052000000	-3.061700000
6	2.013800000	3.592100000	-3.061900000
6	-2.652200000	-4.819100000	2.261400000
6	-2.847300000	4.706400000	2.261400000
6	3.415800000	-3.277900000	-3.543400000
6	-4.456000000	-1.487800000	-3.475800000
6	0.939400000	4.602700000	-3.476100000
6	-1.034500000	-4.139500000	4.029200000
6	-3.279500000	-2.994100000	3.836300000
6	-3.067700000	2.965600000	4.029200000
6	-0.953200000	4.337200000	3.836300000
6	2.043400000	-4.930700000	-2.282900000
6	1.087600000	-3.740900000	-4.297900000
6	-5.330400000	0.532900000	-2.311300000
6	-3.837200000	0.765700000	-4.337600000

6	3.126600000	4.349800000	-2.311700000
6	2.581700000	2.940000000	-4.337700000
1	-3.086000000	-5.557500000	2.920400000
1	-1.894400000	-5.301900000	1.665600000
1	-3.397300000	-4.534800000	1.535900000
1	-4.043600000	-2.665800000	3.146200000
1	-2.976900000	-2.140900000	4.421700000
1	-3.747100000	-3.688000000	4.520000000
1	-0.235100000	-4.608500000	3.473600000
1	-0.609800000	-3.354200000	4.633700000
1	-1.421500000	-4.858300000	4.737200000
1	-3.269900000	5.451300000	2.920400000
1	-3.644400000	4.291500000	1.665600000
1	-2.228600000	5.209600000	1.535900000
1	-3.477100000	3.705400000	4.702100000
1	-2.601800000	2.199200000	4.627600000
1	-3.902100000	2.499500000	3.525100000
1	-1.375300000	5.068700000	4.510400000
1	-0.360200000	3.647400000	4.414900000
1	-0.246700000	4.839600000	3.191200000
1	1.352400000	5.366600000	-4.119200000
1	0.474900000	5.086800000	-2.632100000
1	0.109700000	4.142600000	-3.988300000
1	1.788300000	2.438900000	-4.873700000
1	3.347700000	2.211000000	-4.127700000
1	3.017300000	3.659400000	-5.016200000
1	2.712800000	4.830400000	-1.436700000
1	3.929600000	3.704200000	-1.994200000
1	3.600900000	5.106100000	-2.920700000
1	3.795500000	-4.059700000	-4.185600000
1	4.110900000	-3.141200000	-2.730800000
1	3.471000000	-2.344100000	-4.079500000
1	1.087700000	-2.815200000	-4.855300000
1	0.066000000	-3.969000000	-4.039700000
1	1.416400000	-4.517600000	-4.973500000
1	2.708900000	-4.833400000	-1.437100000
1	1.075200000	-5.233200000	-1.917600000
1	2.384400000	-5.758300000	-2.888200000
1	-5.324200000	-1.512100000	-4.118800000
1	-4.642900000	-2.132000000	-2.631800000
1	-3.642800000	-1.976400000	-3.988000000
1	-6.195500000	0.527700000	-2.958900000
1	-5.169300000	1.548200000	-1.986300000
1	-5.590700000	-0.036000000	-1.430300000
1	-4.711300000	0.759300000	-4.972900000
1	-3.580400000	1.791400000	-4.127200000
1	-3.003000000	0.380100000	-4.906000000

Table S4. The results of LS-analisys of GED data. Calculated and experimental amplitudes of vibrations and their groups

Number of term	i-j	N_i	N_j	r_a	l_{calc}	l_{exp}	Group of amplitude
30	C_m-H	26	32	1.077	0.077	0.084(4)	1
48	O_L-C	17	22	1.268	0.042	0.049(4)	-1
52	C_t- C_m	40	45	1.532	0.053	0.058(3)	2
78	Co-O_L	5	18	1.963	0.069	0.071(2)	-3
102	O_L-O_L	20	19	2.290	0.054	0.057(2)	-3
108	C-C_m	13	48	2.503	0.087	0.090(9)	-4
140	O_L-C_m	10	42	2.881	0.129	0.134(5)	-5
182	Co-C	3	12	3.018	0.093	0.097(5)	-5
198	O_L-O_L	15	9	3.231	0.184	0.188(5)	-5
202	Co-Co	3	5	3.170	0.125	0.129(5)	-5
212	Co-O_L	5	20	3.316	0.152	0.156(5)	-5
222	O_L-C_m	10	48	3.420	0.231	0.235(5)	-5
285	O_L-C	15	12	4.288	0.231	0.212(10)	-6
300	Co-C_m	5	40	4.269	0.102	0.098(10)	-6
366	C-C	22	21	4.935	0.251	0.285(24)	-7
381	Co-C_m	4	48	5.231	0.231	0.239(24)	-7
321	Co-C_m	4	42	4.695	0.165	0.165(7)	-8
325	Co-O_L	3	8	4.582	0.207	0.223(7)	-8
378	O_L-C_m	15	56	5.120	0.472	0.482(14)	-9
390	O_L-C_m	17	42	5.500	0.383	0.394(14)	-9
409	O_L-O_L	20	8	5.410	0.327	0.338(14)	-9
484	O_c-C_m	1	44	5.862	0.165	0.141(50)	10
489	C_m-C_m	26	50	5.864	0.890	0.916(16)	11
573	C-C_m	22	42	6.477	0.467	0.452(20)	12
753	O_L-C_m	19	44	6.862	0.503	0.507(17)	13
812	C_t-C_t	40	37	7.280	0.419	0.423(17)	13
826	Co-C_m	3	49	7.322	0.208	0.212(17)	13
765	C_t-C_m	40	42	7.217	0.599	0.608(75)	14
1052	C_m-C_m	56	51	7.998	0.667	0.302(210)	15
1066	O_L-C	8	39	8.158	0.197	0.215(68)	16
1124	C_t-C_t	24	42	8.240	0.537	0.556(68)	16
1266	O_L-C_m	18	27	8.969	0.246	0.194(60)	17
1421	C_m-C_m	53	44	9.400	0.463	0.411(60)	17
1564	C_m-C_m	56	37	10.781	0.300	0.255(110)	19
1571	C_t-C_m	38	44	10.864	0.243	0.309(144)	20
1704	C_m-H	44	69	12.568	0.296	0.364(450)	21
